# Supplementary material for: Enhancing Microalgae Content in Biocomposites through a Mechanical Grinding Method
Source: Polymers (Basel). 2023 Nov 28;15(23):4557. doi: 10.3390/polym15234557 (PMC10708249; doi:10.3390/polym15234557)
Supplement: Supplementary file 1 [file polymers-15-04557-s001.zip › polymers-2693988-supplementary.pdf]

Supporting Information

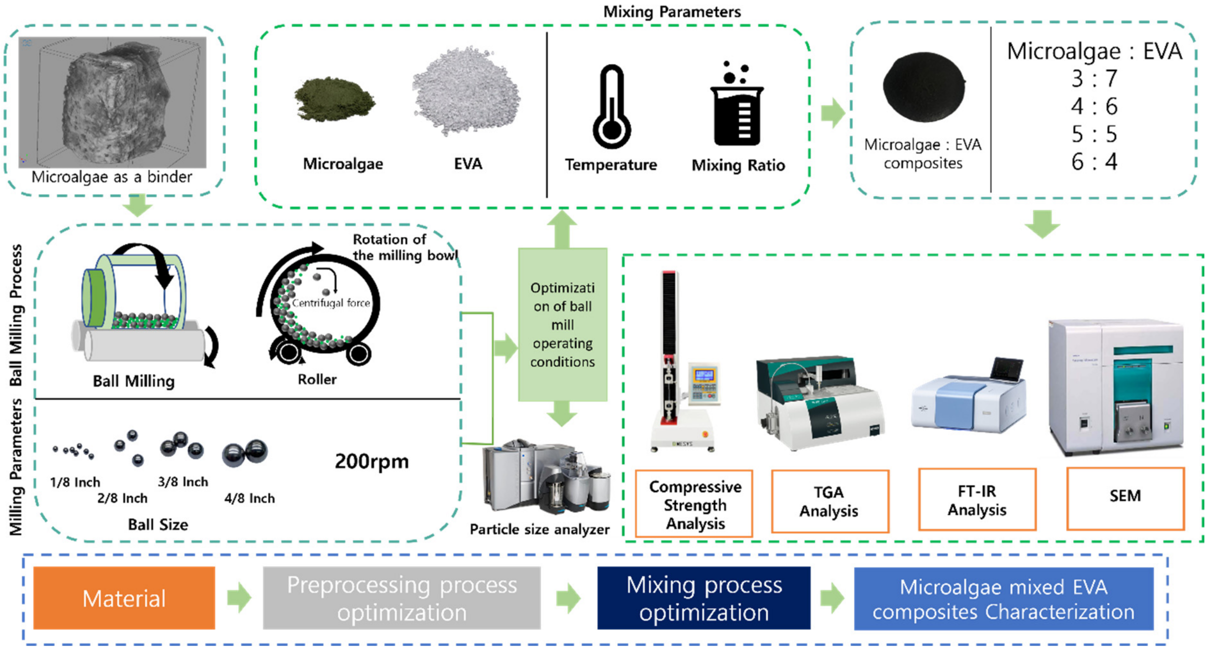

Figure S1. Entire Process of the Experiment: Biocomposite Incorporating Microalgae and EVA.

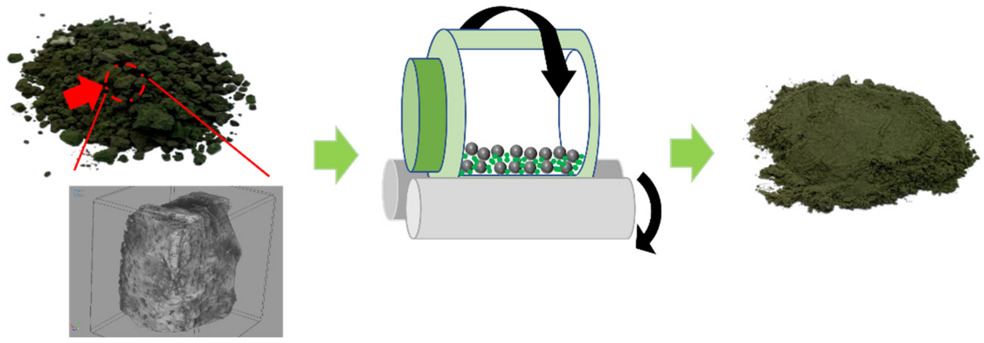

Figure S2. Comparison of Chlorella sp. Before and After Ball Milling.

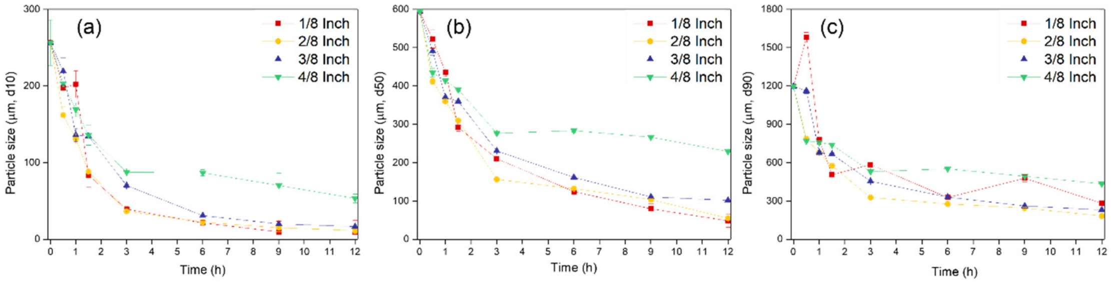

Figure S3. Particle Size Distribution Curves. Fig. S3a represents d10, Fig. S3b represents d50, and Fig. S3c represents d90.
